# Quantification and reduction of cross-vendor variation in multicenter DWI MR imaging: results of the Cancer Core Europe imaging task force

**DOI:** 10.1007/s00330-022-08880-7

**Published:** 2022-06-09

**Authors:** Oliver Lukas Sedlaczek, Jens Kleesiek, Ferdia A. Gallagher, Jacob Murray, Sebastian Prinz, Raquel Perez-Lopez, Evia Sala, Caroline Caramella, Sebastian Diffetock, Nathalie Lassau, Andrew N. Priest, Chikako Suzuki, Roberto Vargas, Tommaso Giandini, Marta Vaiani, Antonella Messina, Lennart K. Blomqvist, Regina G. H. Beets-Tan, Petra Oberrauch, Heinz-Peter Schlemmer, Michael Bach, for the CCE – Imaging Task Force

**Affiliations:** 1 Department of Radiology, German Cancer Research Center (DKFZ), Im Neuenheimer Feld 280, 69120 Heidelberg, Germany; 2 Division of Translational Medical Oncology, National Center for Tumor Diseases Heidelberg (NCT) and German Cancer Research Center (DKFZ), Im Neuenheimer Feld 460, 69120 Heidelberg, Germany; 3 Department of Radiology, University Hospital Heidelberg, Heidelberg, Germany; 4 Department of Radiology, University of Cambridge, Cambridge, UK; 5 Department of Radiology, Vall d’Hebron University Hospital, Barcelona, Spain; 6 Department of Radiology, University of Cambridge and Cancer Research UK Cambridge Centre, Cambridge, UK; 7 Imaging Department, Gustave Roussy, BIOMAPS, UMR1281, INSERM, CEA, CNRS, Université Paris Saclay, Villejuif, Paris, France; 8 Department of Radiology, Addenbrooke’s Hospital, Cambridge, UK; 9 Department of Radiation Physics and Nuclear Medicine, Karolinska University Hospital and Department of Molecular Medicine and Surgery, Karolinska Institutet, Solna, Sweden; 10 Department of Radiology, Karolinska University Hospital and Department of Molecular Medicine and Surgery, Karolinska Institutet, Solna, Sweden; 11 Medical Physics Unit, Fondazione IRCCS Istituto Nazionale dei Tumori, Milan, Italy; 12 Department of Radiology, Fondazione IRCCS Istituto Nazionale dei Tumori, Milan, Italy; 13 Department of Radiology, The Netherlands Cancer Institute, Amsterdam, The Netherlands

**Keywords:** Magnetic resonance imaging, Diffusion-weighted MRI, Radiomics, Multicenter, oncologic studies, Measurement Variability

## Abstract

**Objectives:**

In the Cancer Core Europe Consortium (CCE), standardized biomarkers are required for therapy monitoring oncologic multicenter clinical trials. Multiparametric functional MRI and particularly diffusion-weighted MRI offer evident advantages for noninvasive characterization of tumor viability compared to CT and RECIST. A quantification of the inter- and intraindividual variation occurring in this setting using different hardware is missing. In this study, the MRI protocol including DWI was standardized and the residual variability of measurement parameters quantified.

**Methods:**

Phantom and volunteer measurements (single-shot T2w and DW-EPI) were performed at the seven CCE sites using the MR hardware produced by three different vendors. Repeated measurements were performed at the sites and across the sites including a traveling volunteer, comparing qualitative and quantitative ROI-based results including an explorative radiomics analysis.

**Results:**

For DWI/ADC phantom measurements using a central post-processing algorithm, the maximum deviation could be decreased to 2%. However, there is no significant difference compared to a decentralized ADC value calculation at the respective MRI devices. In volunteers, the measurement variation in 2 repeated scans did not exceed 11% for ADC and is below 20% for single-shot T2w in systematic liver ROIs. The measurement variation between sites amounted to 20% for ADC and < 25% for single-shot T2w. Explorative radiomics classification experiments yield better results for ADC than for single-shot T2w.

**Conclusion:**

Harmonization of MR acquisition and post-processing parameters results in acceptable standard deviations for MR/DW imaging. MRI could be the tool in oncologic multicenter trials to overcome the limitations of RECIST-based response evaluation.

**Key Points:**

• *Harmonizing acquisition parameters and post-processing homogenization, standardized protocols result in acceptable standard deviations for multicenter MR–DWI studies.*

• *Total measurement variation does not to exceed 11% for ADC in repeated measurements in repeated MR acquisitions, and below 20% for an identical volunteer travelling between sites.*

• *Radiomic classification experiments were able to identify stable features allowing for reliable discrimination of different physiological tissue samples, even when using heterogeneous imaging data.*

**Supplementary Information:**

The online version contains supplementary material available at 10.1007/s00330-022-08880-7.

## Introduction

MRI is widely accepted as the imaging modality of choice for noninvasive tumor characterization due to excellent soft tissue contrast resolution. Additionally, the value of functional/physiological imaging in multiparametric MRI is well established, particularly for diffusion-weighted imaging (DWI). DWI is increasingly being used in routine MRI examinations for diagnosis, for staging, and also for therapy response evaluation in cancer patients [1–3]. DWI offers the opportunity to quantify water movement in the tissue microstructure; however, the lack of standardization of DWI acquisition parameters (particularly the “*b*-values”) [4] leads to inadequate comparability between institutions, which is of particular importance in multicenter clinical trials.

Despite the advantages of multiparametric MRI, CT and size-based response evaluation (Response Evaluation Criteria In Solid Tumors/RECIST/RECIST 1.1) [5] remains the accepted outcome measure in oncologic trials despite its inherent limitations [6, 7]. MRI is capable of depicting not only changes in size, but physiological processes such as early changes in tumor cellularity and microenvironment which could complement size criteria.

A response evaluation including such microstructural information would be particularly helpful as many new oncologic therapies, e.g., tyrosine kinase inhibitors [8] and immune checkpoint inhibitors, may not show their efficacy through size reduction alone.

Therefore, an ideal image-based treatment response evaluation method should have the potential to accurately assess and stratify responding and non-responding patients. DWI has shown outstanding performance in the detection of metastases, including in organs considered challenging with standard imaging such as malignant bone marrow [9] or peritoneal infiltration [10]. Changes in tumor cellularity occur earlier than changes in tumor size [11, 12]; therefore, DWI may improve patient care by providing new criteria of response for cancer patients with a noninvasive, radiation-free method to accelerate drug development by allowing earlier readouts of response.

The Cancer Core Europe Consortium (CCE) was established to harmonize cancer research between large European cancer research centers in order to conduct cutting-edge clinical oncologic research. It comprises seven centers. The CCE concept is to form a multi-site virtual cancer institute, which will accelerate the development of new treatments. In this setting, the use of standardized imaging is essential in order to produce harmonized data.

Radiomics is an emerging medical category of image post-processing methods that extracts multiple features from radiographic medical images using data characterization algorithms [13]. These imaging features are considered to be useful for predicting prognosis and therapeutic response, for example, in personalized therapy. Homogeneous imaging data are considered to be a prerequisite for successful application of radiomics [14]; thus, we considered a radiomic evaluation of the data to be a maker for image quality. In turn, stable and yet discriminative radiomics features, even when using heterogeneous imaging data, could be the key to future radiomics studies.

As opposed to previous efforts to standardize acquisition parameters, such as in the German Cancer Consortium (DKTK), in the CCE setting the MR hardware used was produced by various vendors [15, 16].

The aim of this study is to assess and quantify the current variability of MRI parameters (including structural and physiological imaging) in the CCE centers and to quantify the residual variability of a CCE-wide harmonized common MRI protocol aiming to yield (vendor-independent) homogeneous MR imaging results.

## Materials and methods

Data were acquired at the 7 radiology departments participating in the CCE (Cancer Research UK Cambridge Centre, German Cancer Research Center & National Center for Tumor Diseases Heidelberg, Gustave Roussy, Istituto Nazionale dei Tumori, Karolinska Institutet, The Netherlands Cancer Institute, and Vall d’Hebron Institute of Oncology). At all sites, both phantom and volunteer MRI measurements were performed in the scanners planned to be used in CCE studies (Table 1). For all scanners, a core protocol including a single-shot T2-weighted sequence (single-shot T2w) (vendor-specific names: HASTE (Half-Fourier Acquisition Single-shot Turbo spin Echo imaging)/SS-FSE (single-shot fast spin echo)/SS-TSE (single-shot turbo spin echo)) and a DW-EPI sequence including *b*-values 100, 500, and 900 s/mm^2^ was implemented keeping parameters as similar as possible (see Appendix for details).

**Table 1:** MRI scanner types

Center	MRI
A	GE Optima MR450w
B	GE Discovery MR450
C	Philips Achieva dStream
D	Siemens Avanto
E	Siemens Avanto fit
F	Siemens Aera
G	Philips Ingenia

Two volunteer data sets were acquired:

- “Test-Retest”: A volunteer was positioned in the scanner and underwent the core CCE protocol. After initial imaging, the coils were disconnected and reconnected and the volunteer was re-positioned (< 15 min apart) in the scanner and the full procedure was repeated. These volunteers were different between the centers.
- “Volunteer in all scanner”: One of the volunteers undergoing the test-retest design traveled to the other 6 participating centers and there underwent the core CCE protocol.

Each measurement consisted of an unenhanced upper abdominal scan. The volunteer traveling to all centers was identical to the test-retest volunteer of center F, in order to have a reference for the relative comparisons.

A total of 14 + 7 data records (DWI and single-shot T2w) were thus included to investigate the variability of MR measurements in the multicenter setting.

### Hardware

Phantom and volunteer measurements were performed at the seven CCE sites (“A”–“G”). MRI manufacturers are the following: A: GE Optima MR450w, B: GE Discovery MR450, C: Philips Achieva dStream, D: Siemens Avanto, E: Siemens Avanto Fit, F: Siemens Aera, G: Philips Ingenia. The field strength was 1.5 T, except for site C, where a 3-T machine was used. The spine and the body array imaging coils were used.

### MRI acquisition parameters

DWI and single-shot T2w sequences were standardized among the centers. The focus was placed on the quantitative evaluation of the DWI parameters, more precisely the evaluation of the comparability of the measured apparent diffusion coefficient (ADC).

DWI parameters were as follows: field of view (FoV): 340 mm × 293 mm; matrix: 130 × 112 (in-plane resolution 2.6 mm isotropic); slice thickness: 5 mm; TR between 7022 and 8500 ms; TE minimal between 60 and 90 ms; parallel imaging acceleration factor 2; *b*-values 100, 500, and 900 s/mm^2^, with 1, 6, and 23 signal averages for the different *b*-values; diffusion scheme: 3-dimensional diagonal with Siemens Healthcare, 3-in-1 with GE Healthcare, and gradient overplus with Philips Healthcare; receiver bandwidth approximately 2000 Hz/px.

Single-shot T2w imaging (HASTE/SSFSE/SS-TSE): field of view (FoV): 400 mm × 336 mm; matrix: 320 × 256 (in-plane resolution 0.6 mm isotropic); slice thickness: 5 mm; no gap; TR 1000; TE 70; parallel imaging acceleration factor: 2.

### Phantom

A spherical phantom with a diameter of 20 cm filled with an aqueous polyvinylpyrrolidone (PVP) solution and an integrated thermometer were used (HQ Imaging GmbH). The desired ADC was adjusted by the PVP concentration (here: 1600 μm^2^/s at 20 °C). The temperature dependency of the ADC was taken into account by calibration curves [17]. The correction for temperature effects was done in three steps: (1) measure the temperature within the phantom, (2) perform the MRI scan and ADC calculation, and (3) calculate the ADC values at a common standard temperature (here 20 °C) via the use of the calibration curves. In this way, it was not necessary to wait for the phantom to be in thermal equilibrium, which can take hours. This procedure was considered much more practical in a clinical setting than using an ice-water bath.

**Fig. 1:**
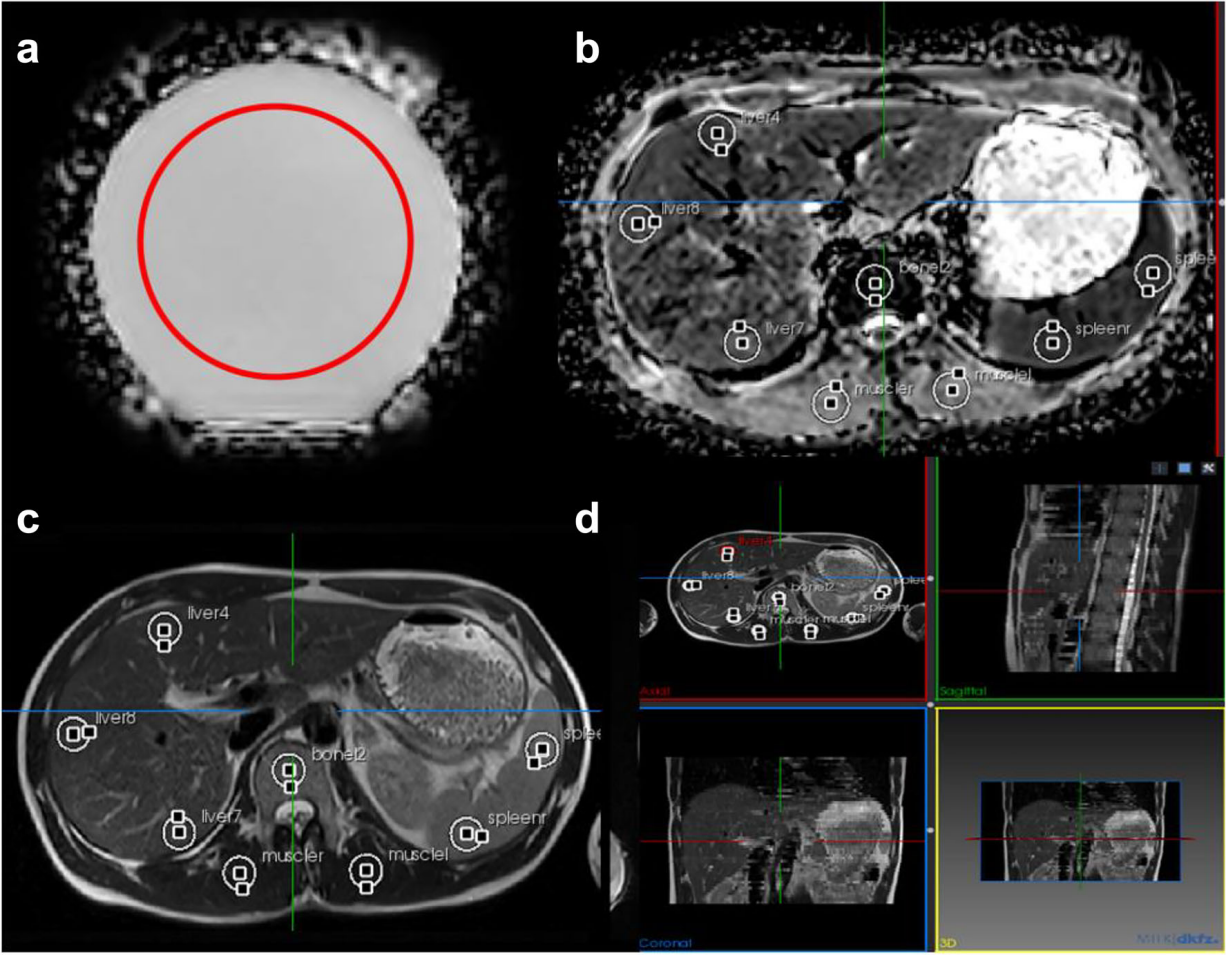
Example of ROIs used in the phantom and volunteer measurements. **a** Automatically drawn ROI (red) in the phantom ADC map and (**b**) manually drawn ROIs in the liver/spleen for ADC determination. **c** Manually drawn ROI in the liver/spleen in single-shot T2w and (**d**) 3D positioning of the ROIs

The mean ADC value at the phantom, its standard deviation, and the correction of temperature effects were performed in an automatically placed ROI of fixed size (see Fig. 1a).

### Volunteer image analysis

Two types of volunteer measurements were performed (see above): in the first setting “test-retest” for an estimation of the intra scanner variability; in the second setting “volunteer in all machines” to test the comparability between centers.

For in vivo evaluations, manually placed circular ROIs were used reflecting the situation in clinical studies.

For both ADC and single-shot T2w values, masks were drawn by a board-certified radiologist in 11 anatomical positions (left and right kidney; liver segments 4, 7, and 8; 2 locations within the spleen, left and right M. Quadratus lumborum, lumbar vertebral body 1 and 2) as illustrated in Fig. 1b–d. The 2D circular masks had a fixed size of 250 mm^2^ and were placed using MITK (Fig. 1) [18].

### Post-processing

ADC maps are usually calculated directly by the scanner (in-line). However, different vendors used different algorithms. This might decrease the comparability between different imaging centers. We therefore compared the ADC values provided directly by the scanner with ADC maps calculated centrally with the same algorithm for all centers. In this algorithm, the standard monoexponential model of diffusion was used, omitting *b* 0. The logarithmic signal decay with respect to the *b*-values was least-square-fitted to a linear function. The slope of the fitted linear curve yields the ADC [19].

### Radiomics

In order to distinguish between the different organs, data were resampled to 1 × 1 × 1 mm^3^. To compensate for vendor-specific factors such as receiver coil sensitivities, patient loading, and receiver gains for the single-shot T2w, we additionally performed histogram matching using 3D slicer [20] with default parameters (10 match points and 128 histogram levels); for each data set (hat (single-shot T2w) = volunteer in all machines, trt_hat1 = Test-retest 1, trt_hat2 = Test-retest 2), site 1 (i.e., DKFZ/center F) served as reference.

Image analysis was performed using custom python scripts. We used PyRadiomics [21] version 2.2.0 to extract 1409 features (see Supplement) for each ROI. To automatically determine a small feature subset (*N* = 15), we employed a two-stage feature selection process. First, we computed the average absolute Pearson correlation for each anatomical position across sites to identify stable features. Features below an arbitrary threshold of 0.75 were disregarded. Second, for the remaining features (100 for ADC/44 for single-shot T2w), we computed the best predictors for the discrimination of the anatomical labels using ANOVA *F*-value scoring as implement in scikit-learn version 0.21.3 [22]. A list of the identified features can be found in Supplement Tables 1 and 2.

Classification was performed using a random forest classifier with 1000 trees, a maximum depth of 10, and default parameters as implemented in scikit-learn otherwise. We performed two different experiments: (a) using each “volunteer in all scanner” data sets, we conducted 10 runs of leave-one-site-out cross-validation for organ prediction based on the selected subset of radiomics features; (b) for the test-retest experiments (ADC and single-shot T2w), we used all sites of the initial acquisition (time point 1) to train the classifier and then predicted the organs of the second acquisition; average results and standard deviation of five runs are reported.

For visualization, we performed dimensionality reduction of the *N* = 15 features using tSNE with default parameters. In addition, we computed the average ROC curves across the 10 repeated runs, the 5 organs, and the 7 investigation sites using scikit-learn version 0.23.2 [22].

The first Wasserstein distance between all pairs of histograms of the anatomical ROIs was computed using SciPy version 1.3.1 employing default parameters [23]. The values were normalized to be in the range between [0,1]. A value of zero indicates identical distributions, whereas higher values reflect dissimilarities of the histograms.

### Statistics

Statistical analysis was performed by using Excel (Office 2016, Microsoft) and R statistical computing software (version 3.0.3; http://www.r-project.org/). ADC variations in a test-retest scenario were assessed according to the Bland-Altman methodology [24, 25]. Mean, relative difference, and standard deviation of the differences of two subsequent measurements on each machine are calculated and depicted in a Bland-Altman plot. The coefficient of variation (CoV) was estimated as CoV = standard deviation / mean (%). A Wilcoxon signed-rank test was performed to assess the variations in ADC values calculated at the MRI device and centrally. The significance level was adjusted according to the number of comparisons that were performed (Bonferroni correction, *p* < 0.007).

## Results

### Phantom: standardized ADC acquisition

**Fig. 2:**
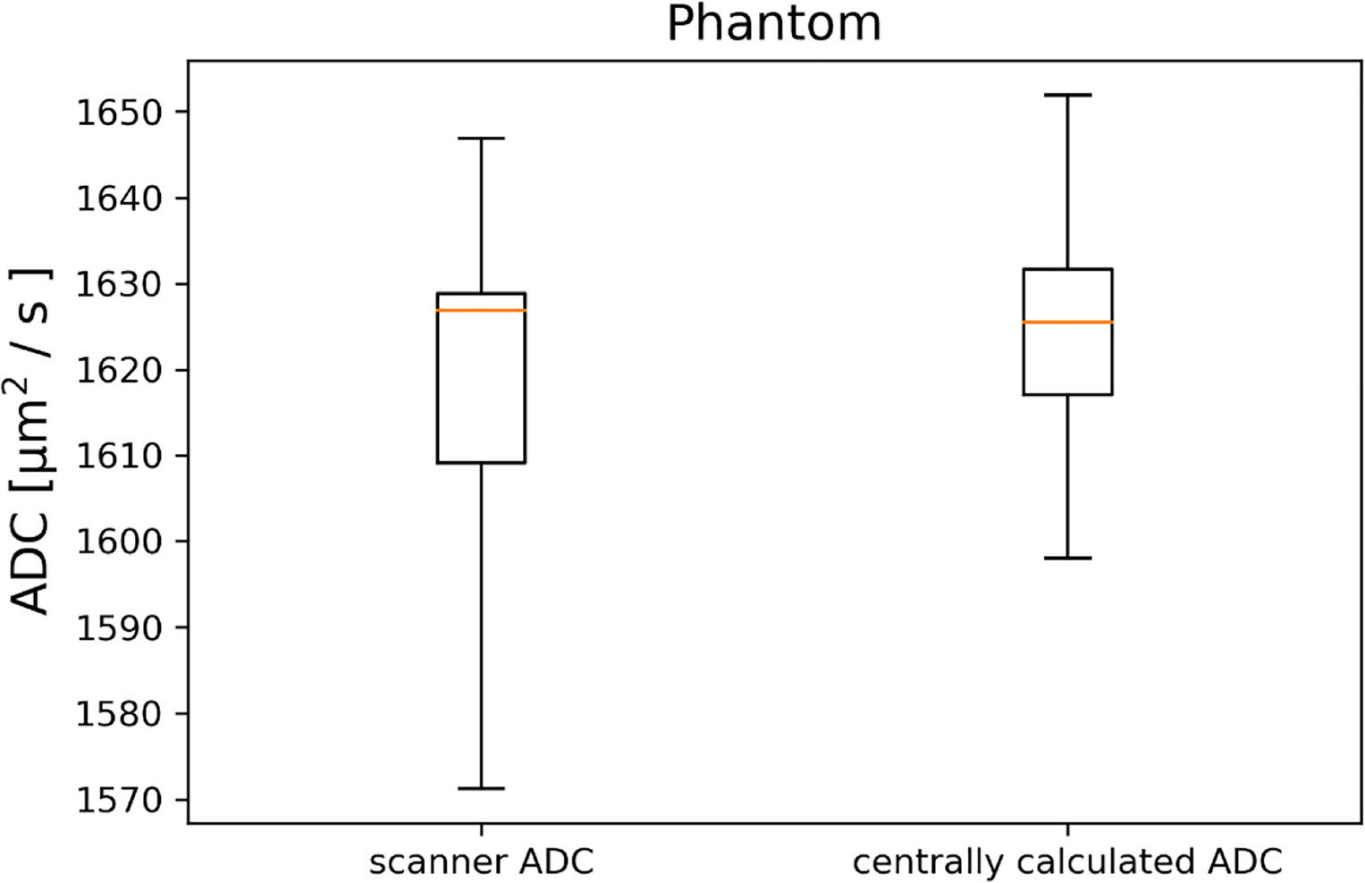
ADC values in the phantom for the seven imaging centers (“A”–“G”), determined with the scanner ADC map and a centrally calculated ADC map. The error bars indicate the standard deviation within the ROI

The result of the ADC phantom measurements in all centers is shown in Fig. 2. The phantom measurements reflect the ADC measurement under ideal conditions: high SNR, no partial volume effects, and no perfusion. The ADC value determined from the in-line calculation on the scanner amounted to 1617 ± 22 × 10^−6^ mm^2^/s (coefficient of variation: 0.0136) and under these conditions, the maximum deviation (i.e., highest measured ADC − lowest measured ADC) in the ADC among the centers was 4%. The comparability was increased further when the common, central post-processing algorithm was used for the ADC calculation. In this case, the ADC value amounted to 1624 ± 16 × 10^−6^ mm^2^/s (coefficient of variation: 0.0099) and the maximum deviation decreased to 2%. However, there was no significant difference in the mean or the standard deviation between the centrally calculated ADC values and the ADC values calculated at the scanner consoles (*p* = 0.06).

### Test-retest ADC

**Fig. 3:**
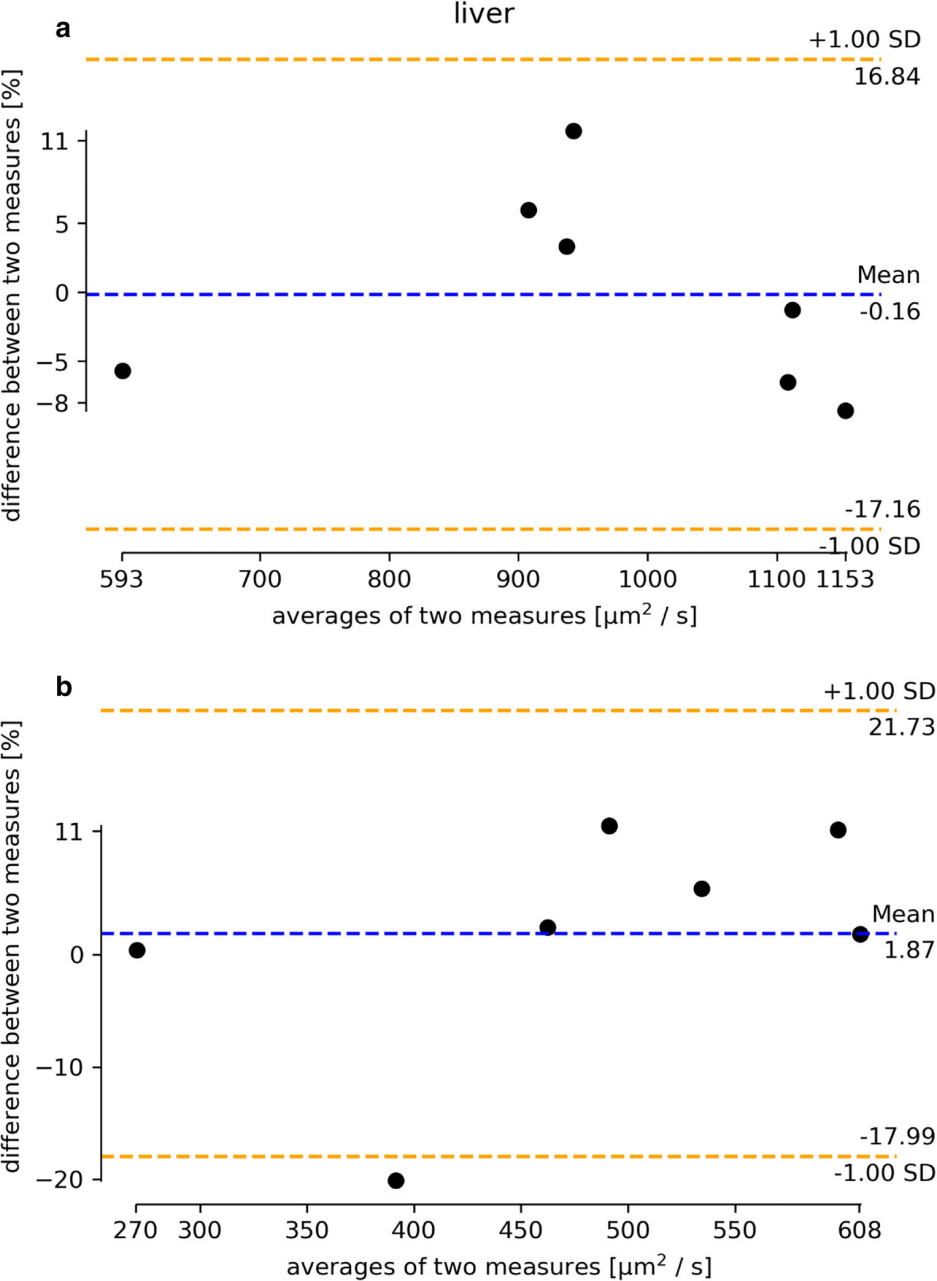
**a** Bland-Altman plot of the relative deviations in ADC values within liver ROIs (averages of the ROI in segments 4, 7, and 8) of the variable volunteers (each liver 3 ROI measurements) at the seven imaging centers. ADC values calculated centrally are depicted (from left to right: sites A, B, G, C, F, E, D). **b** Bland-Altman plot of the gray values of the test-retest for the single-shot T2w within liver ROIs of the variable volunteers for the seven imaging centers (from left to right: sites E, A, D, F, G, B, C)

The Bland-Altman plot in Fig. 3a shows the results of the test-retest measurements for the calculated ADC measured in 3 ROIs of the liver. The mean value of all measurements is depicted by the blue dotted line. The red dotted lines mark one standard deviation of the measurements of the same volunteer at all centers. The deviations in the measured ADC between the first and the second measurements are below 11%.

The comparatively large variations of the ADC (mean: 965 ± 221 × 10^−6^ mm^2^/s) within the liver ROIs reflect the liver structure. The spongy structure of the liver leads to an inhomogeneous ADC map (see Fig. 1). The spleen is characterized by a much more homogeneous ADC map. For other results, see Appendix. Here several results display a smaller standard deviation of the ADC values within an ROI.

Noticeably, there was a large deviation for center “B” between the “in-line” ADC value calculated on the scanner and the centrally calculated ADC value. This was due to the fact that the acquisition of *b* 0 s/mm^2^ was mandatory with the software version of the MR device at the time of data sampling. In contrast at the other centers, ADC measurements contained *b* 100 s/mm^2^ as the lowest B value. The use of *b* = 0 s/mm^2^ leads to an increase in the ADC due to perfusion effects. In the central calculation of the ADC values, these restrictions were not present and the ADC for center “B” was calculated on the basis of the same *b*-values (100, 500, 900 s/mm^2^) as for the other centers. This significantly reduces the maximum deviation and standard deviation of the ADC among the centers (maximum deviation scanner ADC/central calculated: 36/11%; standard deviation scanner ADC/central calculated: 93/35 μm^2^/s) and was therefore applied both in phantom and volunteer measurements.

### Test-retest single-shot T2w

The same test-retest evaluation was performed for the gray levels detected in the single-shot T2w measurement, with the results shown in Fig. 3b.

The variations in gray levels of the single-shot T2w measurements (mean: 480 ± 123) within the liver ROIs are again reflecting inhomogeneous liver structure.

### Volunteer in all machines: ADC

**Fig. 4:**
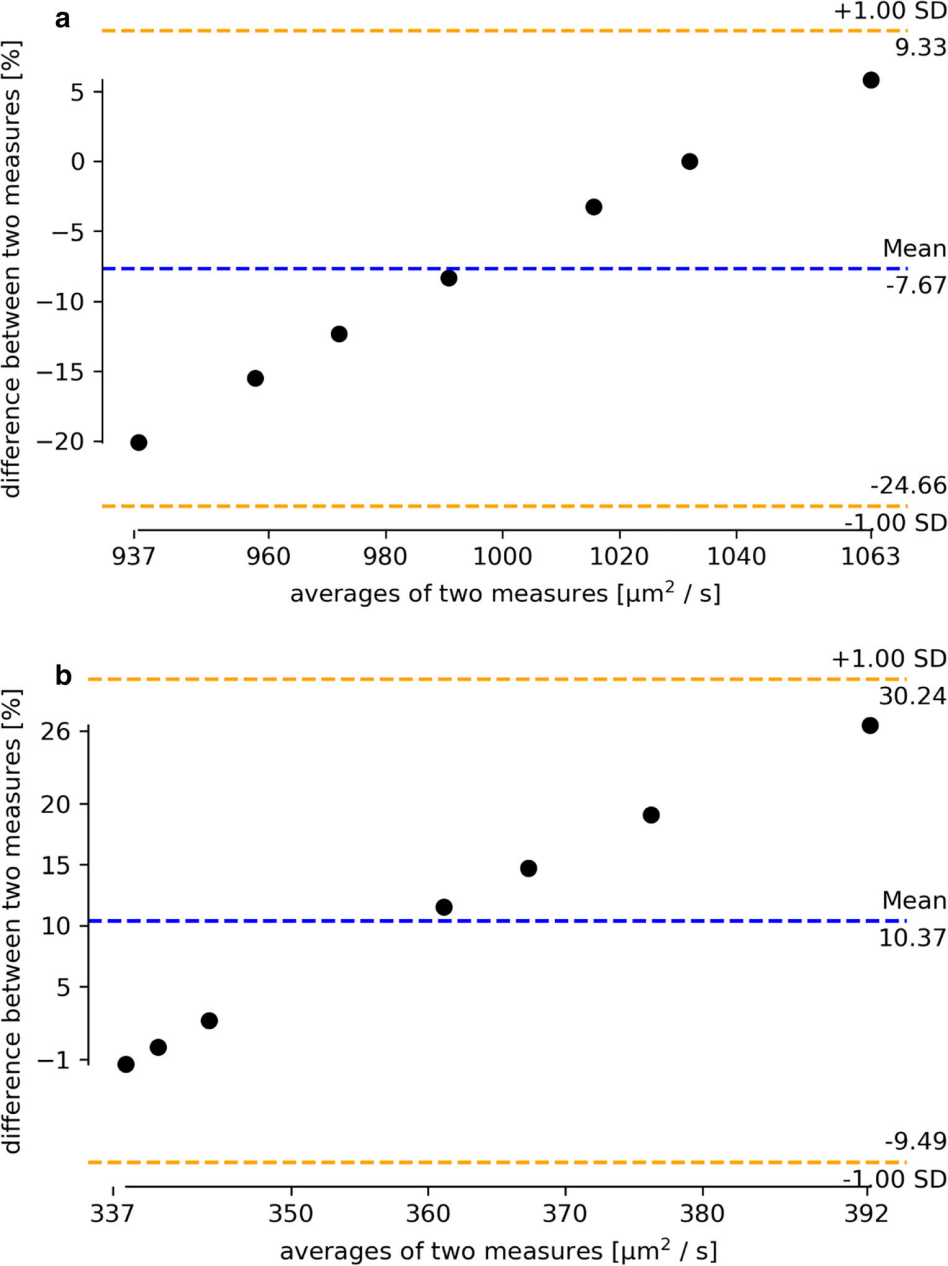
**a** Bland-Altman plot of the relative deviations in ADC values within liver ROIs of an identical volunteer for the seven imaging centers (reference measurement at center F = 0). ADC values calculated centrally are depicted (from left to right: sites C, G, D, A, E, F, B). **b** Bland-Altman plot of the relative deviations of the gray values for the single-shot T2w within (each liver 3 ROI measurements) of an identical volunteer for the seven imaging centers (reference measurement at center F = 0) (from left to right: sites E, F, C, D, B, A, G)

The reference for the “volunteer in all scanner” comparison was the result of the measurements of the travelling volunteer at center “F” and the variations given the results measured at the 6 other participating centers undergoing the core CCE protocol at the various centers. Therefore, the result for center “F” is always the origin “0.” The Bland-Altman plots for ADC and T2w signal intensity are shown in Fig. 4a and b.

### Tissue characterization using radiomic features

#### Experiment 1—identical volunteer at all sites

**Fig. 5:**
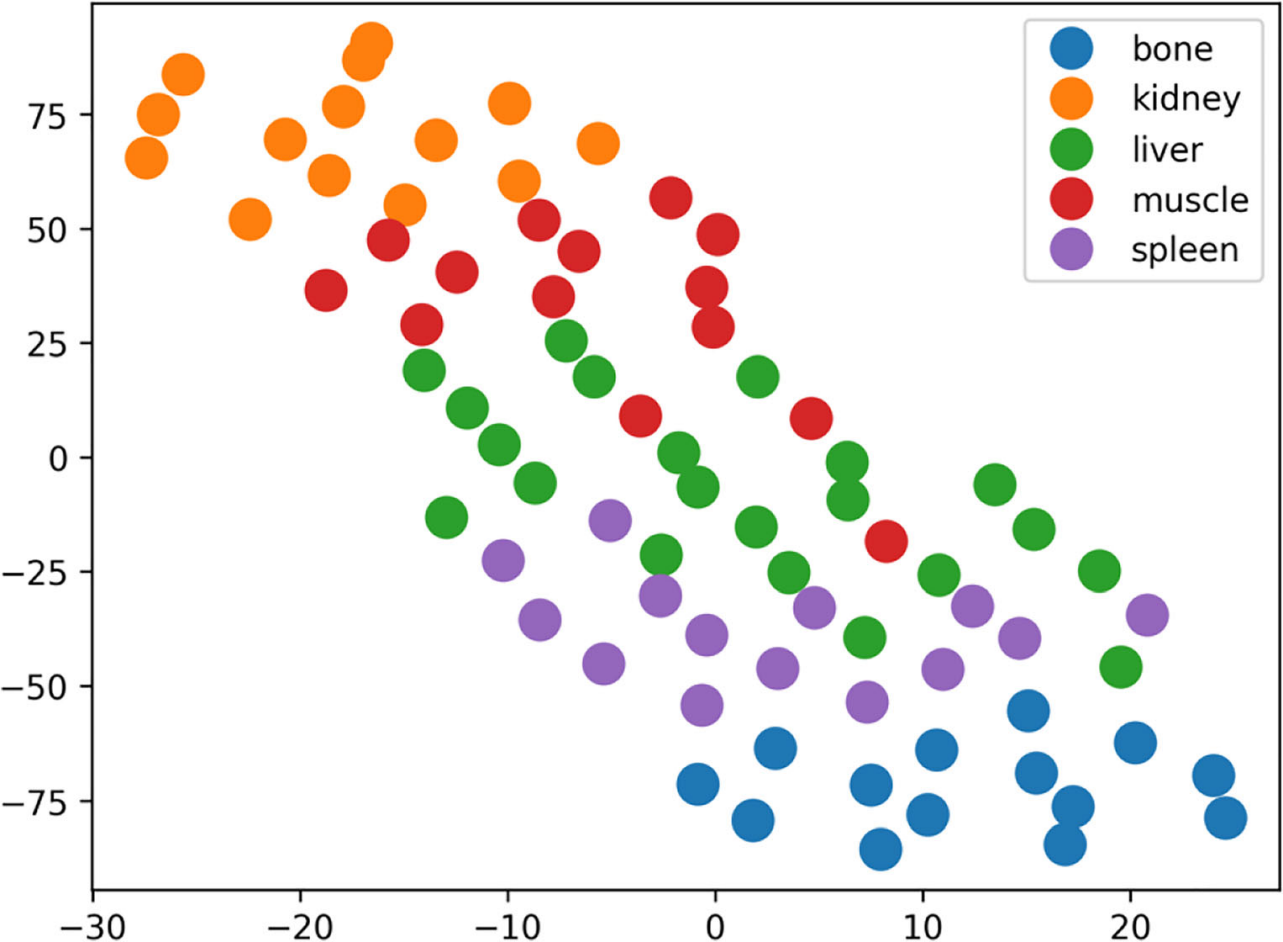
2D-embedding of radiomics features using TSNE. Arbitrary units

A subset of 15 radiomic features were identified as described above. We performed a dimensionality reduction of those features to qualitatively demonstrate the inherent structure within the features characterizing the different anatomical regions. As can be seen in Fig. 5, most of the features extracted from the organ ROIs cluster in distinct regions.

The radiomics feature subset was also used to train a classifier. The goal was to predict the anatomical entities within the imaging data of one site based on training with the remaining sites. This was achieved with an average accuracy of 0.87 for the ADC and 0.75 for the single-shot T2w sequence, respectively. Results are summarized in Table 2 for the ADC and single-shot T2w sequences.

**Table 2:** Classification accuracies and standard deviations for volunteer data training with one-site-leave-out

Site	ADC	Single-shot T2w
A	0.91 (1.11e−16)	0.53 (3.64e−02)
B	0.82 (1.11e−16)	0.55 (1.11e−16)
C	0.75 (4.17e−02)	0.93 (3.64e−02)
D	0.81 (2.77e−02)	0.75 (3.64e−02)
E	1 (0.0)	0.99 (2.73e−02)
F	0.82 (1.11e−16)	0.91 (1.11e−16)
G	1 (0.0)	0.62 (3.64e−02)

Receiver operating characteristic (ROC) curves and area under the curve (AUC) measures (Supplementary Figure in the Appendix) were generated for the classifiers trained on the radiomic feature subset. In addition, the average ROC curves and AUC values over all sites for the ADC and single-shot T2w sequences were computed. A mean AUC of 0.98 (± 0.02) for ADC and of 0.96 (± 0.04) for single-shot T2w was obtained.

#### Experiment 2—test-retest

In a second experiment, we assessed the reproducibility of training a model with features from the first time point for predicting the anatomical entity of the retest (second) time point. This time, the random forest classifier yielded an average accuracy of 0.87 (0.012) for the ADC sequence and of 0.61 (0.007) for the single-shot T2w sequence, respectively.

**Fig. 6:**
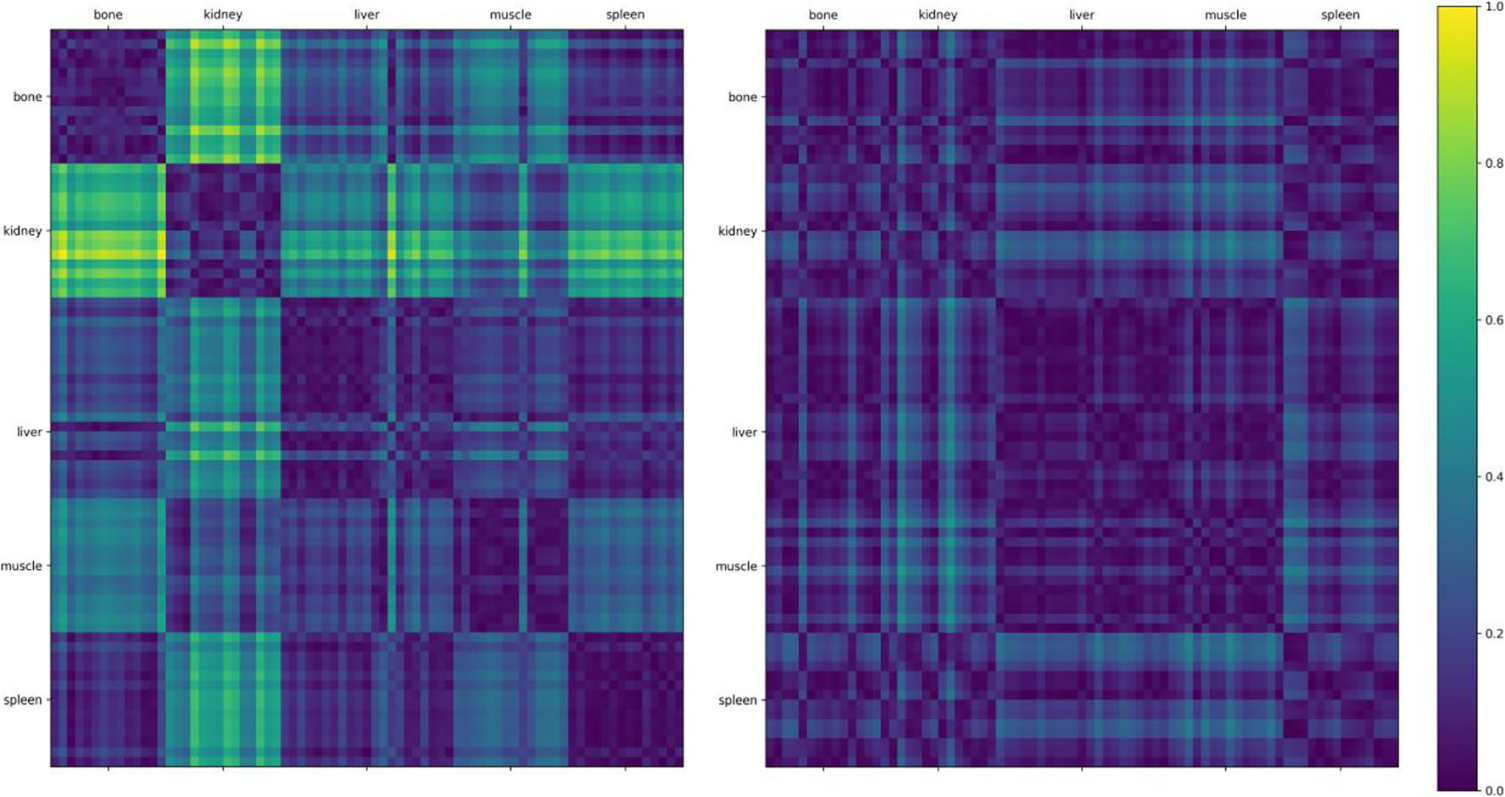
Wasserstein distance between the two test-retest measurements of the seven sites for the ROIs of the different anatomical regions for the ADC (left) and single-shot T2w sequence (right). The smaller the distance, the more similar are the histograms of the anatomical ROIs

For the test-retest data, we also computed the Wasserstein distance between the ROIs of the different anatomical regions. This distance measures the effort it would take to transform one histogram into another. In our experiment, we used this metric to visualize the structure corresponding to the different anatomical ROIs (Fig. 6). In comparison to the distances of the single-shot T2w histograms, the distances of the test-retest ADC histograms allow for a better visual delineation of the different anatomical regions.

## Discussion

Our data show that after harmonization of acquisition parameters and post-processing homogenization, standardized protocols result in acceptable standard deviations for ROI-based evaluations, particularly for DWI/ADC measurements, paving the way for multicenter clinical trials with large patient cohorts.

The technical and biological variations in an MR examination, including the repeated shimming, patient placement, sequence measurement, and ROI selection as well as mild temperature or hydration state differences of the volunteer/patient, are reflected by the scan-rescan data. Here the total measurement variation does not to exceed 11% for ADC and is below 20% for our structural imaging surrogate, the single-shot T2w (Figs. 3 and 4). If we compare the differences in MR imaging with consecutive ROI measurements undertaken in an identical volunteer travelling between sites, 20% for ADC and < 25% for single-shot T2w were not exceeded.

For phantom measurements, differences in ADC can be reduced to values as low as 2% (coefficient of variation: 0.0136). Here variation can be reduced significantly by omitting *b*-value 0 and applying standardized central post-processing. Obviously in humans, perfusion effects can lead to a strong dependence between the *b*-values used and the resulting ADC [26, 27]. Some of the residual deviations may be introduced by vendor-specific reconstruction algorithms.

Oncologic multicenter trials mostly use the objective tumor response rate (ORR) to treatment or progression-free survival (PFS) to evaluate antitumor responses, which incorporate the change in tumor burden. While size measurements according to RECIST do not differ between MRI and CT (except for the lung), the additional physiological and soft tissue information using MRI in oncologic studies is largely neglected. This is partly due to the high complexity and variability in quantitative readouts that is expected.

In addressing the limitations of RECIST-based response evaluation, it is important to quantify the discrepancies seen in real-world clinical studies. For interobserver variability, the detection of new lesions may be divergent between local readers and central-blinded readers by more than 50%, while the tumor burden measurements differed by around 18% between the readings [28]. Similar conclusions were drawn in a meta-analysis on RECIST-based tumor burden measurements [29], showing the relative interobserver difference in single lesion measurements to be 20% and more.

Thus, the relative interobserver differences seen for anatomic assessments in the multicenter MR measurement alone of > 20% seem to be in keep [29]. The fact that the variances seen in a single volunteer do exceed the ones seen in the test-retest setting is not unexpected for readers used to look at medical images of different vendors; however, being able to quantify them and the restricted amount of the variance detected is promising.

In the clinical setting, the lower availability of MR measurement slots and the at least 3-fold longer scan times have to be discussed against quicker scan times of CT. Here the clinical benefit of MRI has explicitly to be shown particularly for elderly patients being stable over several re-staging intervals. However, for the initial therapeutic phase and the situation in clinical studies, early response predictions supported by functional MR data seem of great value.

In our study, the single-shot T2w was used as an example for structural images. Objective parameters for image quality are largely lacking, and SNR estimates might be misleading. However, gray level variations did not relevantly exceed those seen in DWI/ADC.

Regarding the signal variations seen for MRI in humans, it has to be considered that measurement differences do occur partly due to the advantage of MRI: the high soft contrast in MRI as compared to CT. In a recent publication using manually segmented ROIs in a multicenter whole-body-MRI (3Tesla, single vendor) setting investigating healthy volunteers, repeatability and reproducibility were quantified [16]. Here 95% confidence intervals on repeatability and reproducibility suggested ADC signal changes for the abdominal findings above 16–45% (liver 35%) to be clinically significant. The slightly higher variances found might be partly due to the use of whole-body protocols and the on average higher field strength used, leading to increased artifacts particularly at the lung/liver and lung/spleen interface. While there was a systematic error in comparing 1.5-T and 3-T machines with regard to ADC measurements of the bone marrow in a recent publication [30], no such effect was seen in our data, but there was only a single 3-T system used in the participating centers.

In radiomics, the extraction of features is considered to be sensitive to inhomogeneous image quality [31, 32]. In a recent publication exploring the stability of radiomic features from T2-weighted MRI of cervical cancer, mainly shape features were found to be stable [33]. In our experiments, lacking shape characteristics, the classification (both experiments), and visual separation (Fig. 6) work better for ADC than for single-shot T2w imaging. This is plausible, since ADC is considered to be “more” quantitative. Classical radiomics features, e.g., mean, were identified. This is also reasonable and similar to how humans would make distinctions when measuring and comparing ROIs.

Of course, in our study the introduction of radiomic features was a feasibility application. We aimed at demonstrating that, when using our harmonized acquisition techniques and the identical volunteer across sites, the imaging data can be used for the development of quantitative imaging biomarkers. This is an artificial setting and we do not claim that a valid, generalized radiomics model was obtained. For this purpose, more samples (patients) would be necessary. Nevertheless, in both classification experiments, we were able to identify stable features that allow for a reliable discrimination of five different physiological tissue samples—albeit using very limited training data. Furthermore, using a test-retest scenario, we demonstrated the similarity of our measured signals across sites. This lays the groundwork for determining radiomics features from pathological tissue alterations and will aid in future assessment of multicenter oncological studies.

## Conclusion

Structural oncologic endpoints are relevantly influenced by scan variability and by the individual reading of the images. Thus, additional variations added through multicenter designs have to be minimized to facilitate the detection of treatment effects. In this context, MRI could be beneficial since it adds physiological endpoints to anatomic assessments alone. It is mandatory to harmonize acquisitions as MRI is only a qualitative signal and the data quality can be expected to be even worse in real-world scenarios with patients. Yet, the results of this study seem to be encouraging for the multicenter use of MRI for detecting signal variances comparable to the standard readouts in oncology. From these signals, derived radiomics features are an additional tool that support the characterization of lesions and their response to treatment.

Further efforts in the CCE collaboration will include contrast-enhanced images and dynamic contrast-enhanced MRI (DCE MRI)–time series to evaluate the full range of oncologic imaging.

## Supplementary information

ESM 1 (DOCX 958 kb)

## Abbreviations

ADC: Apparent diffusion coefficient
CCE: Cancer Core Europe Consortium
CT: Computed tomography
DCE: Dynamic contrast-enhanced MRI
DWI/DW-EPI: Diffusion-weighted imaging
HASTE: Half-Fourier Acquisition Single-shot Turbo spin Echo imaging
ORR: Objective tumor response rate
PFS: Progression-free survival
PVP: Polyvinylpyrrolidone
RECIST: Response Evaluation Criteria In Solid Tumors
SS-TSE: Single-shot turbo spin echo
SS-FSE: Single-shot fast spin echo

## Declarations

### Guarantor

The scientific guarantor of this publication is Oliver Sedlaczek.

### Statistics and biometry

No complex statistical methods were necessary for this paper.

### Methodology

• prospective

• experimental

• multicenter study
