# Supplementary material for: Quantification and reduction of cross-vendor variation in multicenter DWI MR imaging: results of the Cancer Core Europe imaging task force
Source: Eur Radiol. 2022 Jun 9;32(12):8617–28. doi: 10.1007/s00330-022-08880-7 (PMC9705481; doi:10.1007/s00330-022-08880-7)
Supplement: Supplementary file 1 — (DOCX 958 kb) [file 330_2022_8880_MOESM1_ESM.docx]

**Appendix :**


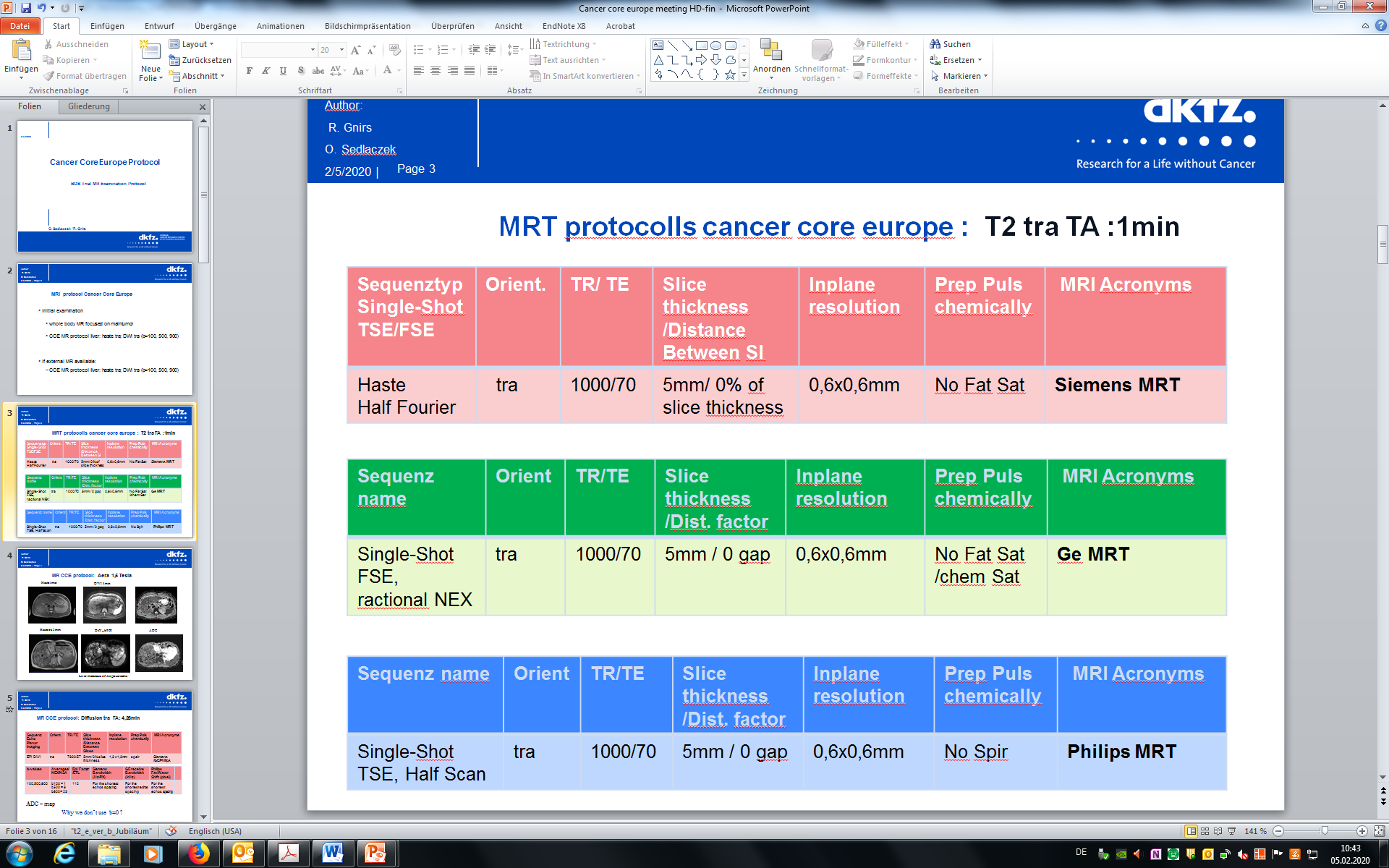


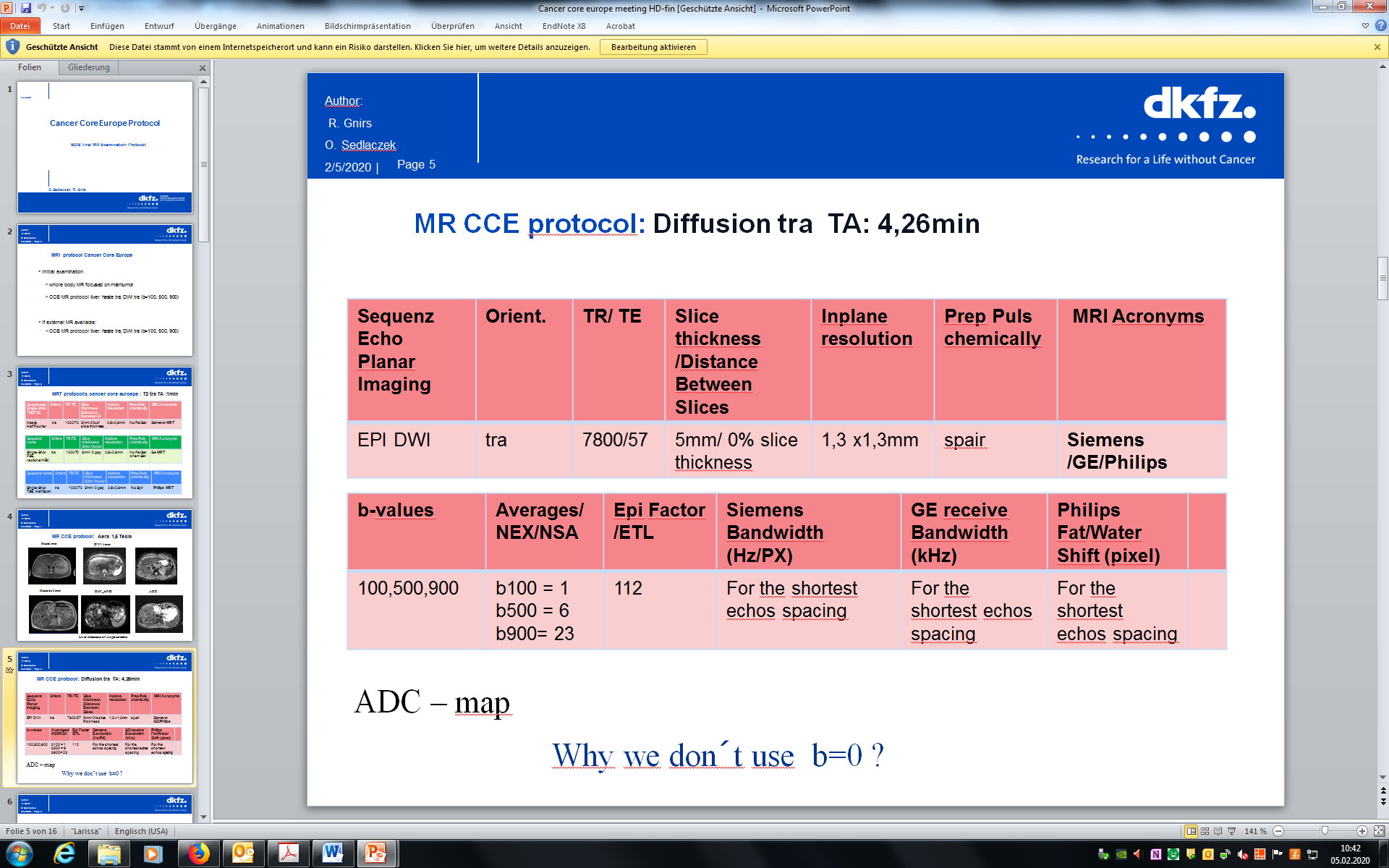


Further Figures Volunteer at all machines:


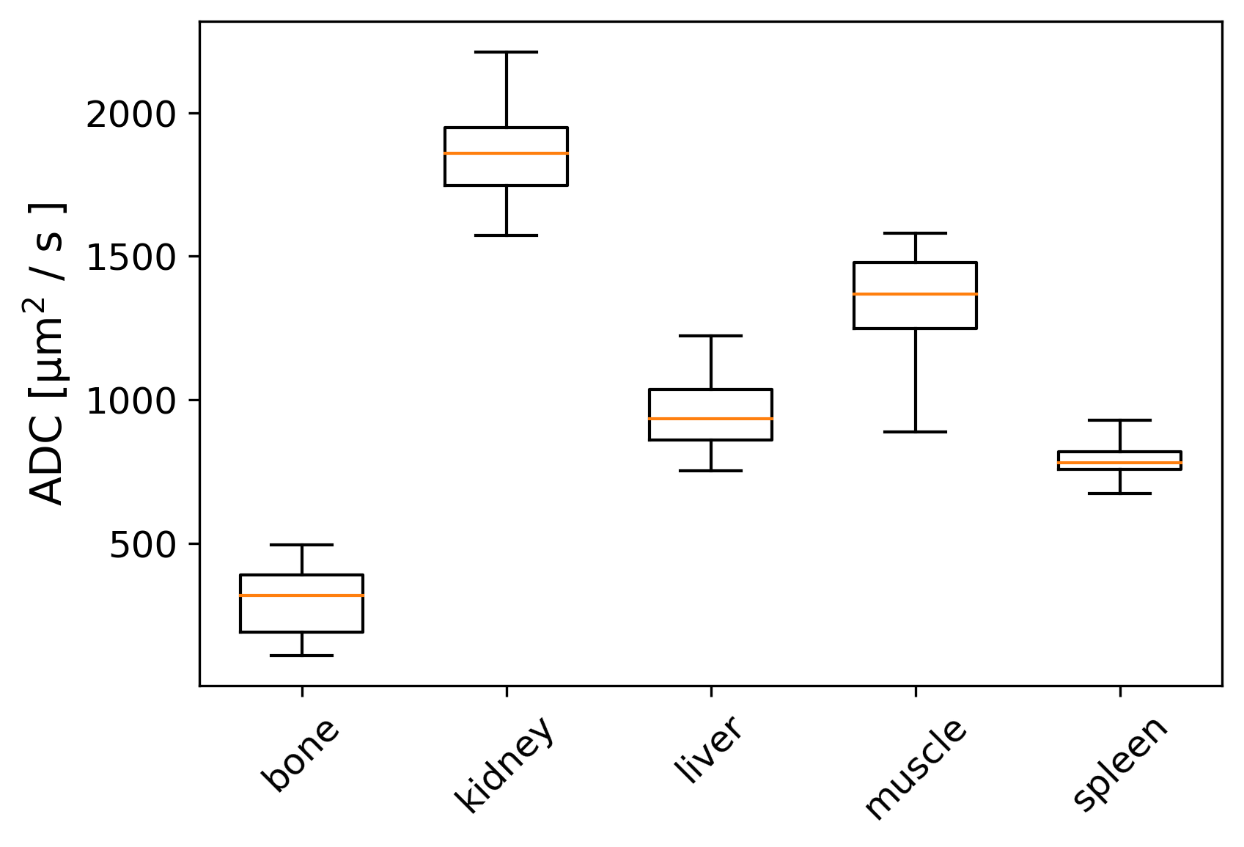


*ADC values for the pooled organ ROIs (averages of the ROIs in organs) of an identical volunteer for the seven imaging centers. ADC values calculated centrally are depicted.*


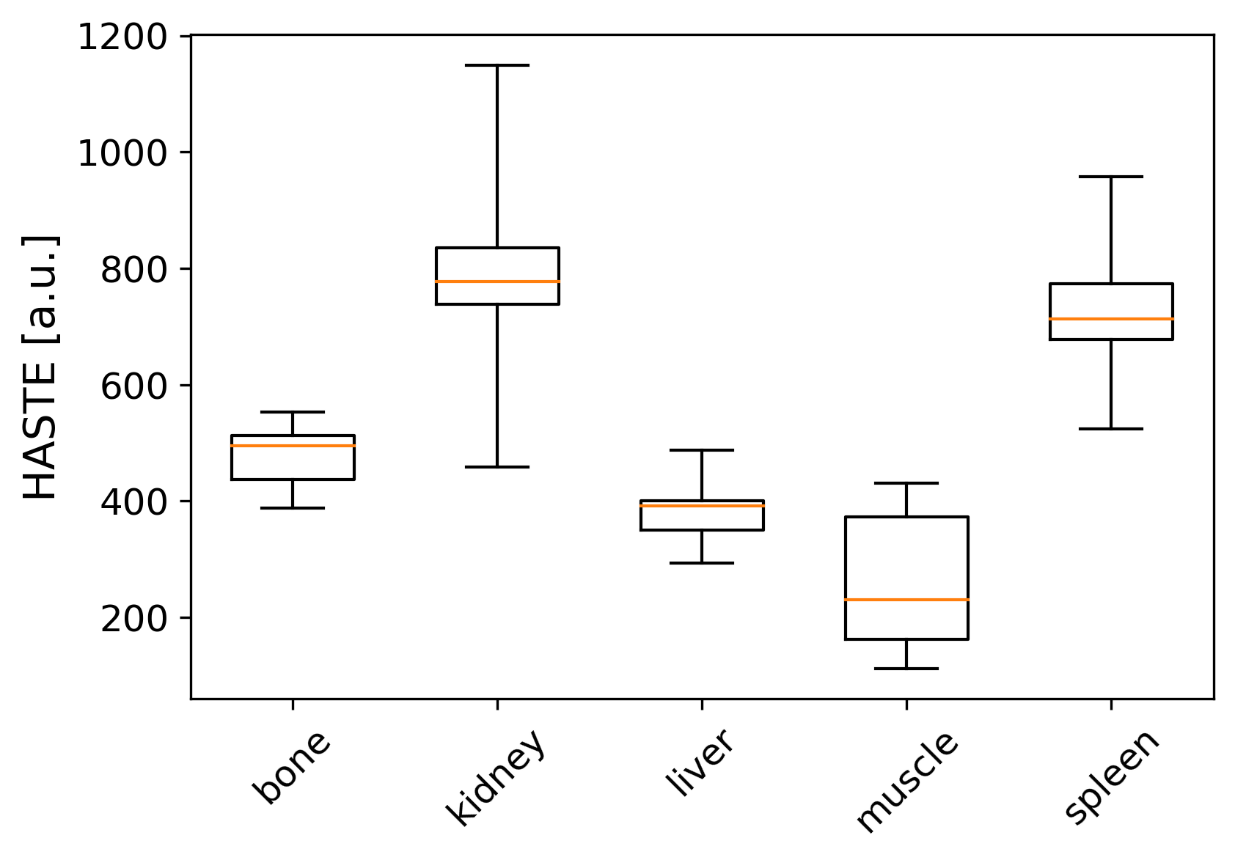


*Single-shot T2w gray-levels for the pooled organ ROIs (averages of the ROIs in organs) of an identical volunteer for the seven imaging centers. ADC values calculated centrally are depicted.*

Supplement Table 1 – 15 Radiomics Features Selected for ADC Sequence Random Forest Model

| Radiomics Feature | F-value | p-Value |
| --- | --- | --- |
| original_firstorder_Mean | 205.38 | 1.43e-38 |
| original_firstorder_Median | 202.05 | 2.46e-38 |
| original_firstorder_RootMeanSquared | 193.22 | 1.07e-37 |
| wavelet-LLL_firstorder_Median | 186.36 | 3.51e-37 |
| wavelet-LLL_firstorder_90Percentile | 145.37 | 1.08e-33 |
| original_firstorder_TotalEnergy | 144.29 | 1.37e-33 |
| original_firstorder_Energy | 144.29 | 1.37e-33 |
| original_firstorder_10Percentile | 143.9 | 1.50e-33 |
| original_firstorder_90Percentile | 141.74 | 2.43e-33 |
| wavelet-LLL_firstorder_10Percentile | 109.84 | 7.15e-30 |
| wavelet-LLL_firstorder_Mean | 107.12 | 1.55e-29 |
| square_gldm_LowGrayLevelEmphasis | 68.55 | 8.30e-24 |
| square_firstorder_Entropy | 47.16 | 2.08e-19 |
| square_glcm_JointEntropy | 46.25 | 3.44e-19 |
| square_glrlm_RunLengthNonUniformity | 45.71 | 4.65e-19 |

Supplement Table 2 – 15 Radiomics Features Selected for ADC Sequence Random Forest Model

| Radiomics Feature | F-value | p-Value |
| --- | --- | --- |
| wavelet-LLL_firstorder_10Percentile | 94.57 | 6.82e-28 |
| wavelet-LLL_firstorder_Minimum | 89.31 | 3.78e-27 |
| wavelet-LLL_firstorder_Median | 89.18 | 3.94e-27 |
| wavelet-LLL_firstorder_Mean | 84.81 | 1.76e-26 |
| wavelet-LLL_firstorder_RootMeanSquared | 83.5 | 2.77e-26 |
| original_firstorder_Minimum | 80.31 | 8.68e-26 |
| original_firstorder_10Percentile | 77.78 | 2.21e-25 |
| original_firstorder_Median | 71.42 | 2.58e-24 |
| wavelet-LLL_firstorder_TotalEnergy | 70.87 | 3.22e-24 |
| wavelet-LLL_firstorder_Energy | 70.87 | 3.22e-24 |
| original_firstorder_Mean | 68.38 | 8.90e-24 |
| wavelet-LLL_firstorder_90Percentile | 66.92 | 1.63e-23 |
| original_firstorder_RootMeanSquared | 66.89 | 1.65e-23 |
| square_firstorder_Median | 65.73 | 2.71e-23 |
| square_firstorder_Minimum | 63.91 | 5.93e-23 |

Supplement – Extracted Radiomics Features

The 1409 radiomics features result from the combination of 15 filters (exponential, gradient, lbp-2D, logarithm, original, square, squareroot, wavelet-HHH, wavelet-HHL, wavelet-HLH, wavelet-HLL, wavelet-LHH, wavelet-LHL, wavelet-LLH, wavelet-LLL) with individuals features that can be categorized into 7 feature types (shape, firstorder, gldm, glcm, glrlm, ngtdm, glszm). For a given filter to feature type combination, the available individual features may vary. The full list of individual features across features types is: 10Percentile, 90Percentile, Autocorrelation, Busyness, ClusterProminence, ClusterShade, ClusterTendency, Coarseness, Complexity, Contrast, Correlation, DependenceEntropy, DependenceNonUniformity, DependenceNonUniformityNormalized, DependenceVariance, DifferenceAverage, DifferenceEntropy, DifferenceVariance, Elongation, Energy, Entropy, Flatness, GrayLevelNonUniformity, GrayLevelNonUniformityNormalized, GrayLevelVariance, HighGrayLevelEmphasis, HighGrayLevelRunEmphasis, HighGrayLevelZoneEmphasis, Id, Idm, Idmn, Idn, Imc1, Imc2, InterquartileRange, InverseVariance, JointAverage, JointEnergy, JointEntropy, Kurtosis, LargeAreaEmphasis, LargeAreaHighGrayLevelEmphasis, LargeAreaLowGrayLevelEmphasis, LargeDependenceEmphasis, LargeDependenceHighGrayLevelEmphasis, LargeDependenceLowGrayLevelEmphasis, LeastAxisLength, LongRunEmphasis, LongRunHighGrayLevelEmphasis, LongRunLowGrayLevelEmphasis, LowGrayLevelEmphasis, LowGrayLevelRunEmphasis, LowGrayLevelZoneEmphasis, MCC, MajorAxisLength, Maximum, Maximum2DDiameterColumn, Maximum2DDiameterRow, Maximum2DDiameterSlice, Maximum3DDiameter, MaximumProbability, Mean, MeanAbsoluteDeviation, Median, MeshVolume, Minimum, MinorAxisLength, Range, RobustMeanAbsoluteDeviation, RootMeanSquared, RunEntropy, RunLengthNonUniformity, RunLengthNonUniformityNormalized, RunPercentage, RunVariance, ShortRunEmphasis, ShortRunHighGrayLevelEmphasis, ShortRunLowGrayLevelEmphasis, SizeZoneNonUniformity, SizeZoneNonUniformityNormalized, Skewness, SmallAreaEmphasis, SmallAreaHighGrayLevelEmphasis, SmallAreaLowGrayLevelEmphasis, SmallDependenceEmphasis, SmallDependenceHighGrayLevelEmphasis, SmallDependenceLowGrayLevelEmphasis, Sphericity, Strength, SumAverage, SumEntropy, SumSquares, SurfaceArea, SurfaceVolumeRatio, TotalEnergy, Uniformity, Variance, VoxelVolume, ZoneEntropy, ZonePercentage, ZoneVariance.


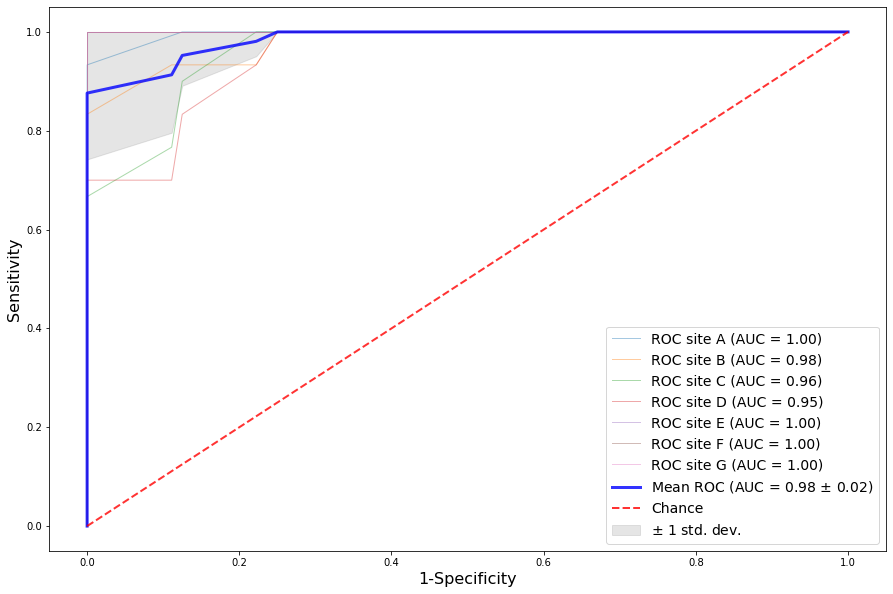

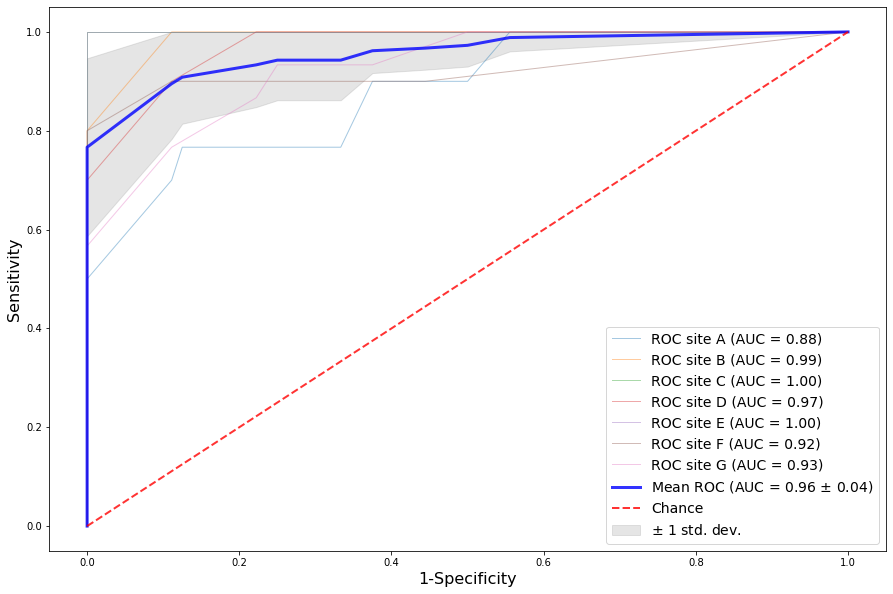


**ROC curves and AUC values for classifier of radiomic features for ADC and single-shot T2w sequences**
